# Supramolecular recognition on the surface of two-dimensional polyketones

**DOI:** 10.1038/s41598-026-41144-8

**Published:** 2026-04-17

**Authors:** Sanaz kazemi, Mohsen Adeli, Mohammad Nemati

**Affiliations:** Department of Organic Chemistry, Faculty of Chemistry, Lorestan University, Khorramabad, Iran

**Keywords:** 2D polymer, Polyketone, Friedel–Crafts, Host–guest interactions, Molecular recognition, Chemistry, Materials science

## Abstract

Two-dimensional polymers constitute an emerging class of materials with structurally defined, atomically thin architectures that impart unique optical, electronic, and host–guest properties. Here, we report the synthesis of a crystalline two-dimensional polyketone (2D-PK) via a Friedel–Crafts acylation reaction at a solid–liquid interface on aluminum foil. This interfacial approach yields micrometer-sized sheets composed of highly ordered, stacked layers, as confirmed by SEM, TEM, AFM, and HRTEM analyses. Spectroscopic and diffraction studies—including characteristic shifts in the carbonyl stretching band in IR spectra, sharp solid-state NMR resonances, and well-defined PXRD reflections—further confirm the formation of a crystalline 2D network with an interlayer spacing of 3.54 Å. Beyond structural characterization, 2D-PK exhibits selective supramolecular interactions with organic dyes in aqueous media. Among the investigated analytes, methyl green (MG) shows a markedly stronger affinity for 2D-PK, manifested by pronounced fluorescence enhancement and red-shifted emission, along with distinct changes in NMR, DLS, BET surface area, and UV–Vis absorption profiles. These host–guest interactions arise from a synergistic combination of electrostatic attraction, confinement within polymeric cavities, and charge-transfer-assisted binding, as supported by experimental data and computational simulations. Overall, this study demonstrates that structurally ordered two-dimensional polyketones function as efficient and selective molecular recognition platforms capable of modulating fluorescence responses at low analyte concentrations. These findings highlight the promise of 2D-PKs as versatile materials for chemical sensing, supramolecular chemistry, and future optoelectronic applications.

**Supplementary Information:**

The online version contains supplementary material available at 10.1038/s41598-026-41144-8.

## Introduction

Polymerization at interfaces offers an effective strategy to spatially confine monomers and control the morphology of macromolecules^1^. In particular, two-dimensional (2D) polymerization at solid–liquid interfaces enable the growth of extended polymer networks by assembling small building blocks on smooth substrates^2,3^. Among various polymerization strategies, Friedel–Crafts reactions have emerged as a powerful tool for constructing porous organic polymers, reminiscent of classical Davankov resin synthesis^4,5^. This method is particularly valuable for generating aryl ketone linkages, which are broadly useful in organic materials chemistry^6,7^. Recent advances have extended Friedel–Crafts chemistry to the synthesis of covalent organic frameworks (COFs) and other 2D materials^8,9^. While aluminum-based Lewis acids are the most common catalysts, Brønsted acids have also been investigated to further develop this reaction to the materials science area^10^. For instance, hypercrosslinked phenyl-based porous polymers have been synthesized via one-step Friedel–Crafts polymerization, and nitrogen-enriched conjugated microporous polymers have been accessed using methanesulfonic acid as a catalyst^11^. Due to their permanent porosity and structural regularity, COFs are highly suited for host–guest molecular recognition. Their defined pore geometries provide cavities for guest inclusion, enabling applications in chemical sensing and separation^12–14^. Fluorescence-based detection, in particular, has been demonstrated using COFs that respond to analyte via enhanced emission^15,16^. This concept has been extended to guest-adaptive COFs with dynamic pores capable of distinguishing small molecules, gases, and even enantiomers^17–19^. Similarly, 2D materials with well-defined structures offer exceptional potential for selective molecular recognition, especially when assembled via covalent or noncovalent approaches^20,21^. Metal confinement in 2D membranes has also yielded promising systems for gas separation and molecular sensing^22–24^. Methylene Green (MG) is a positively charged dye widely used in biological imaging and DNA staining^25,26^. Owing to its relevance in biomedical applications, developing host materials capable of recognizing and modulating the fluorescence of MG is of significant interest. In this work, we report the interface-assisted synthesis of a crystalline two-dimensional polyketone (2D-PK) via Friedel–Crafts polymerization between 1,3,5-benzenetricarbonyl trichloride and mesitylene in dichloroethane. The resulting 2D-PK sheets, with lateral dimensions in the micrometer range, exhibited selective host–guest interactions with MG, resulting in a fluorescence signal absent in the free dye. This system demonstrated the potential of 2D-PKs as molecular recognition platforms for sensing applications.Materials, methods, and experimental details related to the synthesis of 2D-PK are explained in the supplementary information.

## Materials and methods

The materials, methods, and experimental details regarding the synthesis of materials, along with additional information on the characterizations, are provided in the supplementary information.

## Results

**Fig. 1:**
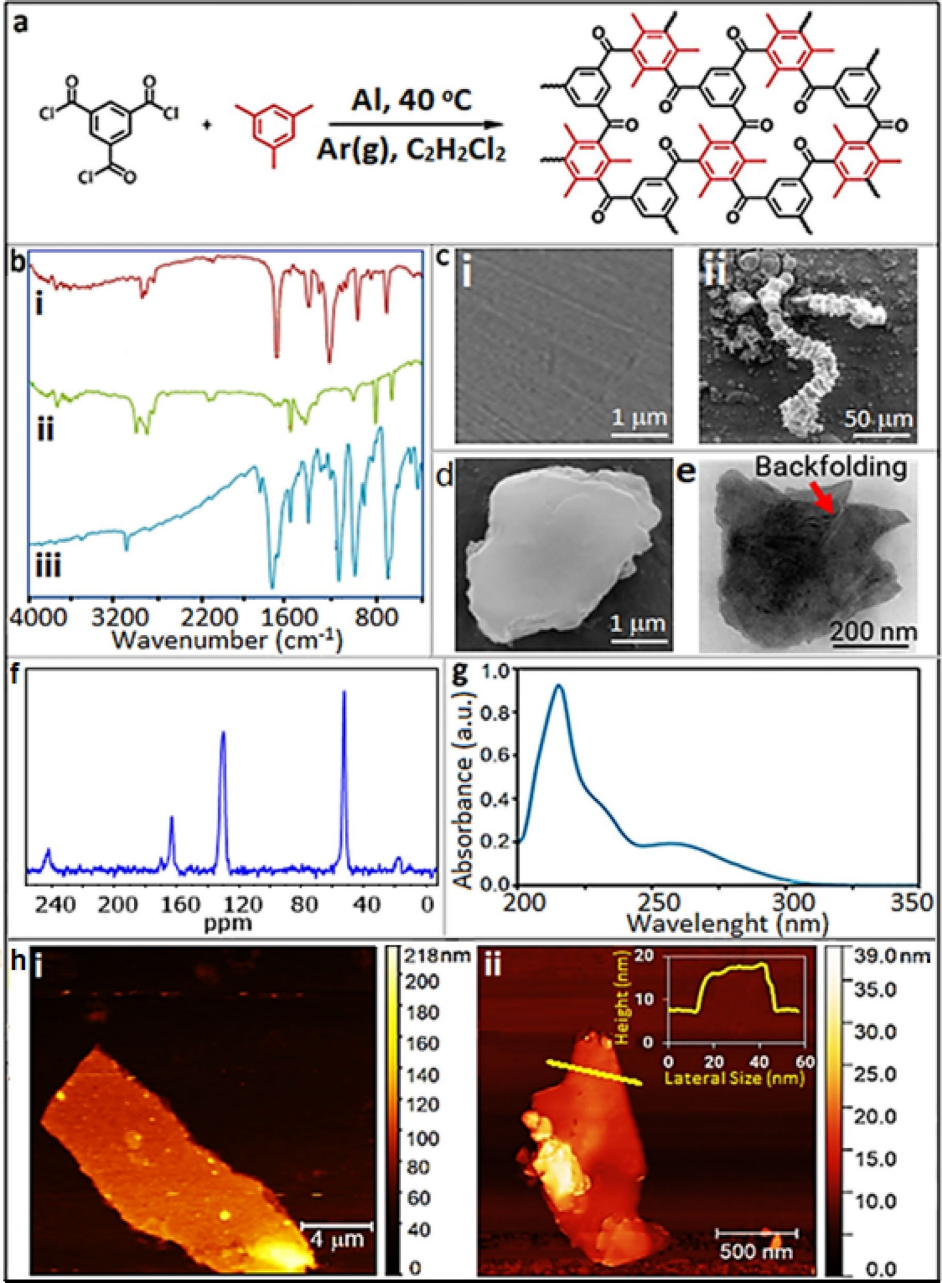
(**a**) Schematic illustration of the synthesis of the two-dimensional polyketone (2D-PK) at a solid/liquid interface via Friedel–Crafts acylation. (**b**) IR spectra of (i) 2D-PK, (ii) mesitylene, and (iii) BTC. (**c**) SEM images of the aluminum foil before (i) and after (ii) the Friedel–Crafts reaction. (**d**) SEM image of 2D-PK sheets after separation from the aluminum foil and purification, revealing layered structures with lateral dimensions of several micrometers. (**e**) TEM image of 2D-PK, showing stacked layers with well-defined edges and visible back-folding. (**f**) CP-MAS NMR spectrum of 2D-PK, showing distinct signals corresponding to aromatic rings and carbonyl functional groups. (**g**) UV–Vis absorption spectrum of 2D-PK, highlighting characteristic absorption peaks. h) AFM image of 2D-PK indicating layered structures with several micrometers lateral sizes and around 8 nm thickness.

Two-dimensional polyketone (2D-PK) was synthesized on aluminum foil via a Friedel–Crafts acylation reaction between 1,3,5-benzenetricarbonyl trichloride (BTC) and mesitylene (Fig. 1a). In the IR spectrum of 2D-PK, characteristic absorption bands at 2950 cm^−1^ and 1257 cm^−1^ correspond to aliphatic C–H and C–O stretching vibrations, respectively. Notably, the carbonyl stretching band of BTC, initially observed at 1761 cm^−1^, shifts to 1731 cm^−1^ after polymerization, indicating successful conjugation of the monomers at the solid–liquid interface (Fig. 1b).

The surface morphology of the aluminum foil before and after the reaction was examined by scanning electron microscopy (SEM). Following polymerization, the initially smooth aluminum surface becomes densely covered with layered structures resembling elongated strands (Fig. 1c, d, and S1), suggesting that polymer growth initiates at the aluminum surface and proceeds in a layer-by-layer manner. The resulting material was subsequently detached from the foil, thoroughly washed, and purified for further characterization.

SEM images of the isolated material reveal sheet-like morphologies composed of multiple stacked layers with well-defined edges, confirming successful polymerization at the solid–liquid interface (Fig. 1c). Transmission electron microscopy (TEM) further supports the layered architecture, showing stacked sheets with clear edges and occasional back-folding features (Fig. 1e).

The^13^ C solid-state cross-polarization magic-angle spinning (CP-MAS) nuclear magnetic resonance (NMR) spectrum of 2D-PK displays distinct resonances at 132 ppm and 53 ppm, which are assigned to aromatic carbons and methyl groups, respectively. In addition, signals at 170 ppm and 165 ppm are attributed to ketone carbonyl groups along the polymer backbone and carbonyl functionalities likely located at the sheet edges (Fig. 1f).

The optical properties of 2D-PK were evaluated using UV–vis absorption and fluorescence spectroscopy. The UV–vis spectrum of 2D-PK exhibits absorption maxima at 211 nm and 260 nm, which are assigned to π–π* and n→π* transitions, respectively. Notably, 2D-PK shows no significant fluorescence emission in the range of 300–800 nm (Fig. 1g).

Atomic force microscopy (AFM) images reveal flat, sheet-like structures with an average thickness of approximately 8 nm, which is attributed to the presence of stacked layers within the 2D-PK architecture (Fig. 1h and Figure S3).

The composition and elemental distribution of 2D-PK were analyzed by energy-dispersive X-ray spectroscopy (EDX). As expected, carbon and oxygen were the dominant elements detected, with elemental ratios closely matching the calculated composition. A trace amount of aluminum was also observed at a negligible level, likely originating from the aluminum substrate used during synthesis (Figure S2).

**Fig. 2:**
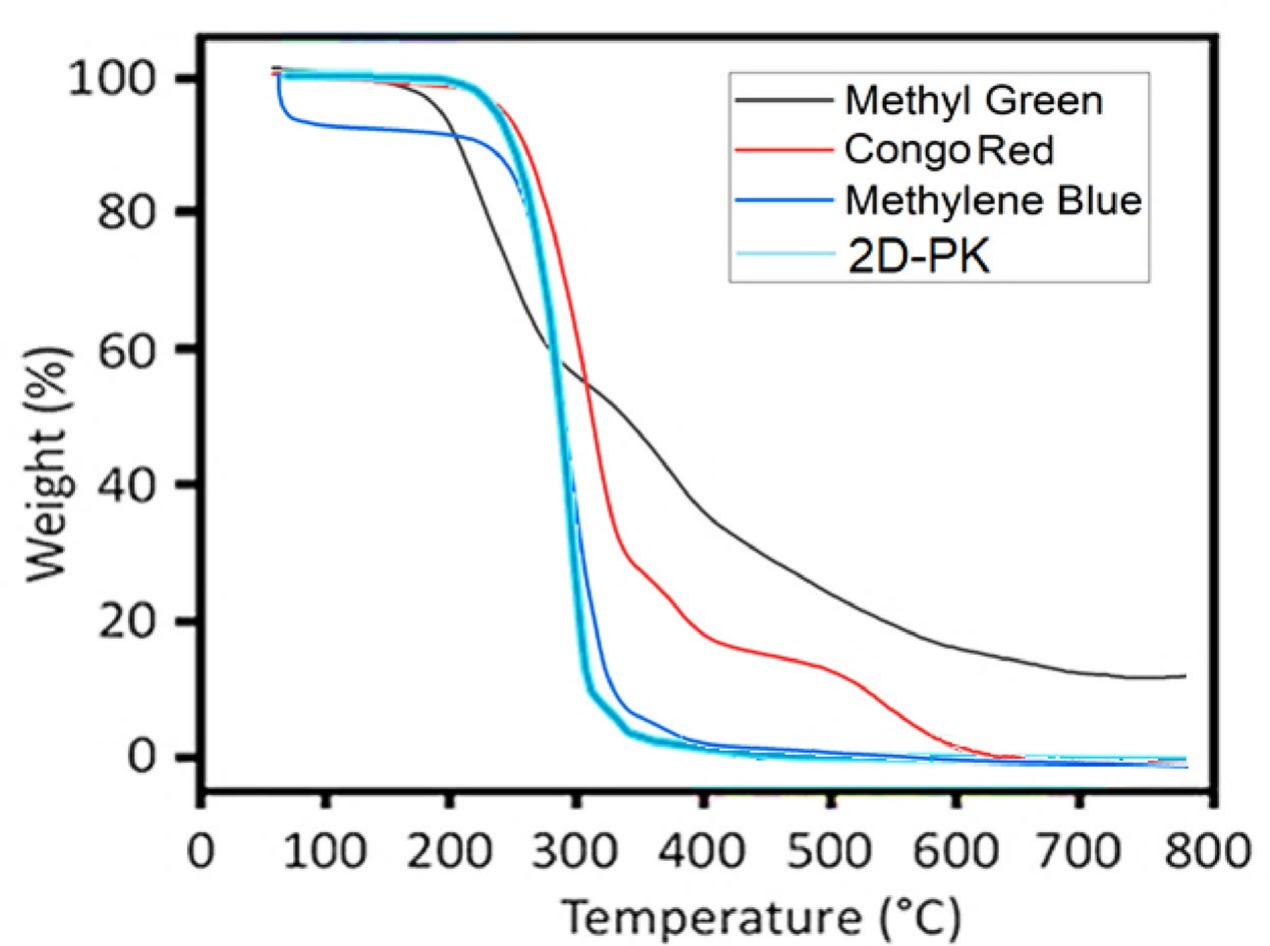
TGA thermogram of the synthesized 2D-PK, 2D-PK/MB, 2D-PK/CR and 2D-PK/MG were evaluated by thermogravimetric analysis (TGA).

The thermal behavior of 2D-PK was investigated by thermogravimetric analysis (TGA), which shows a sharp decomposition event occurring between 250 and 310 °C (Fig. 2). In the presence of methylene blue and Congo red, the primary decomposition profile of 2D-PK remained largely unchanged. In contrast, interaction with methyl green resulted in a significantly broadened weight-loss profile extending from approximately 200 to 650 °C, indicating a distinct interaction between MG and 2D-PK compared to the other dyes.

The sharp and well-resolved resonances observed in the solid-state NMR spectra, together with the narrow decomposition temperature range, indicate that 2D-PK possesses a well-defined structure with a low density of defects.

**Fig. 3:**
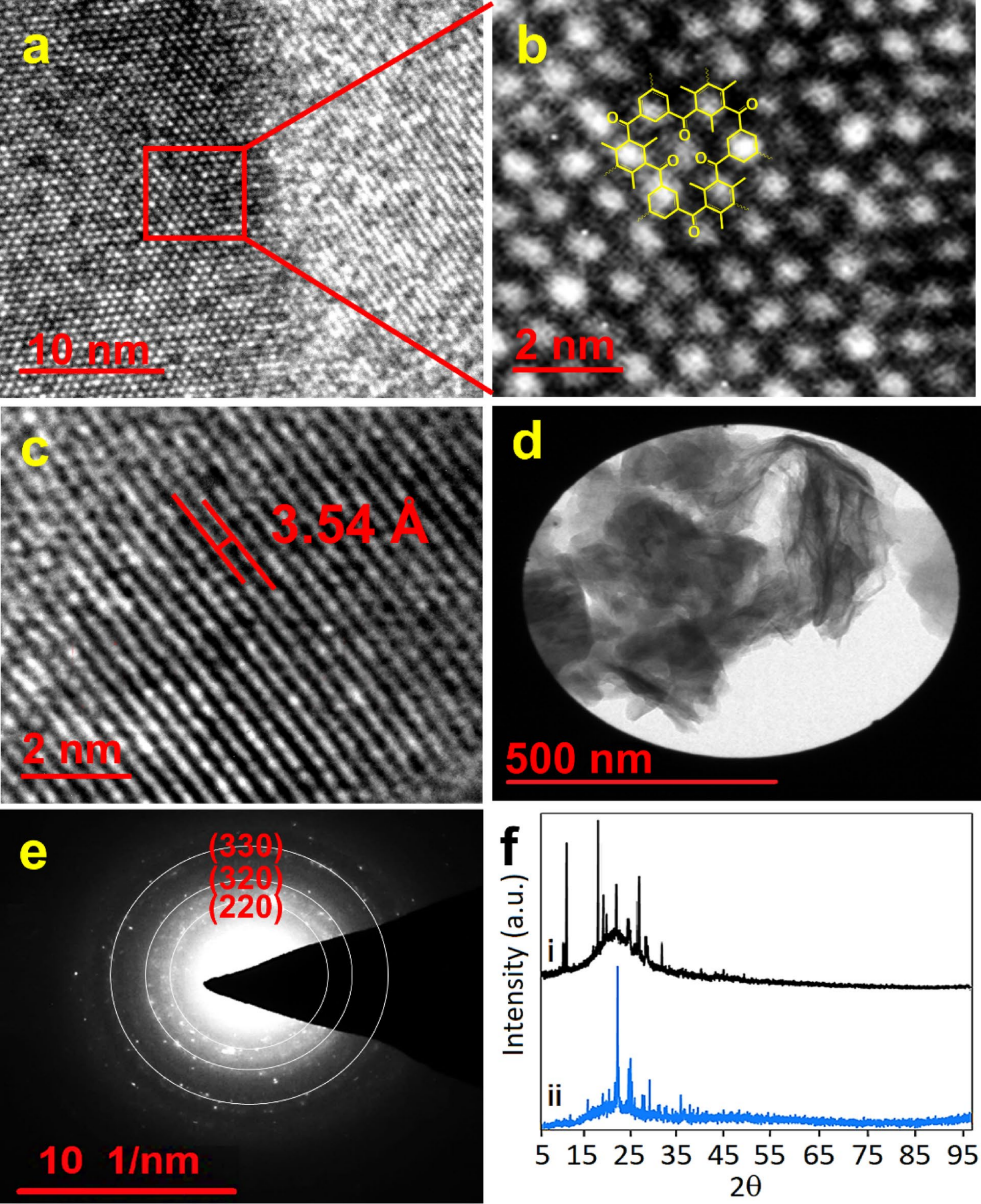
(**a**, **b**) HRTEM images of 2D-PK showing crystalline domains with various orientations. At higher magnification, a highly regular hexagonal structure is observed. (**c**) HRTEM image of a single crystalline domain, revealing stacked layers with an interlayer spacing of 3.54 Å. (**d**, (**e**) The selected-area electron diffraction (SAED) patterns displaying concentric rings corresponding to different crystalline planes, indicating multiple orientations of crystallites. (**f**) XRD diffractogram of 2D-PK (i) and BTC (ii) showing sharp peaks attributed to the crystalline structure of the synthesized material.

Further insight into the atomic structure of 2D-PK was obtained using high-resolution transmission electron microscopy (HRTEM). The analysis reveals crystalline, layered architectures exhibiting a highly regular hexagonal network, consistent with the proposed chemical structure shown in Fig. 3a. The HRTEM images clearly show aromatic rings interconnected to form a porous two-dimensional framework (Figs. 3a–c).

The interlayer spacing was measured to be 3.54 Å, in excellent agreement with the value derived from X-ray diffraction (XRD) analysis (Fig. 3c). Selected-area electron diffraction (SAED) patterns display concentric diffraction rings corresponding to the (220), (310), (311), (320), (330), and (430) crystallographic planes (Fig. 3d and e). This diffraction pattern indicates the presence of multiple crystalline domains with different orientations, consistent with the polycrystalline nature observed in the HRTEM images (Fig. 3d).

Powder X-ray diffraction (PXRD) further confirms the crystalline nature of 2D-PK. Sharp reflections at 2θ = 17.79°, 18.79°, 21.86°, 24.50°, 26.75°, 28.36°, and 31.63° were indexed to the (430), (330), (320), (320), (311), (310), and (220) planes, respectively. The reflection at 2θ = 28.32° was assigned to the (800) plane and corresponds to an in-plane diffraction feature. Notably, the peak at 2θ = 26.75° is attributed to π–π stacking interactions between adjacent layers (Fig. 3f). The interlayer distance calculated using Bragg’s equation is 3.54 Å, in excellent agreement with the HRTEM measurements.

**Fig. 4:**
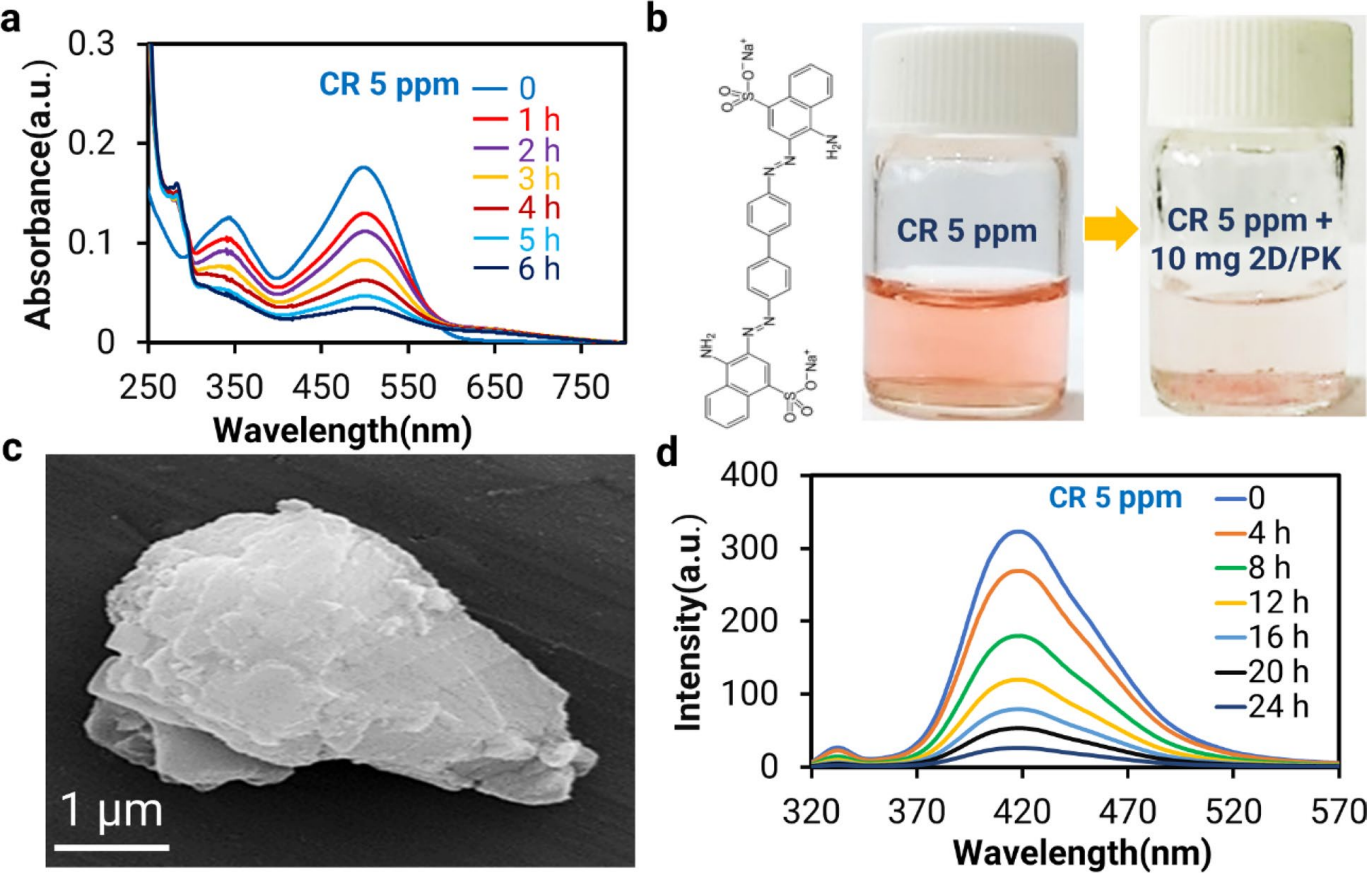
(**a**) UV–Vis spectra of aqueous solutions of CR with 5 ppm concentration before and after incubation with 2D-PK at different time frames. (**b**) Aqueous solutions of CR (10 mg/L) after incubation with 2D-PK for 2 min (left) and 1 h (right). (**c**) ). SEM image of 2D-PK sheets after incubation with CR for 24 h. (**d**) The fluorescence spectra of aqueous solution of CR (5 ppm) at λ_ex_ = 310 nm before and after incubation with 2D-PK at different time frames.

**Fig. 5:**
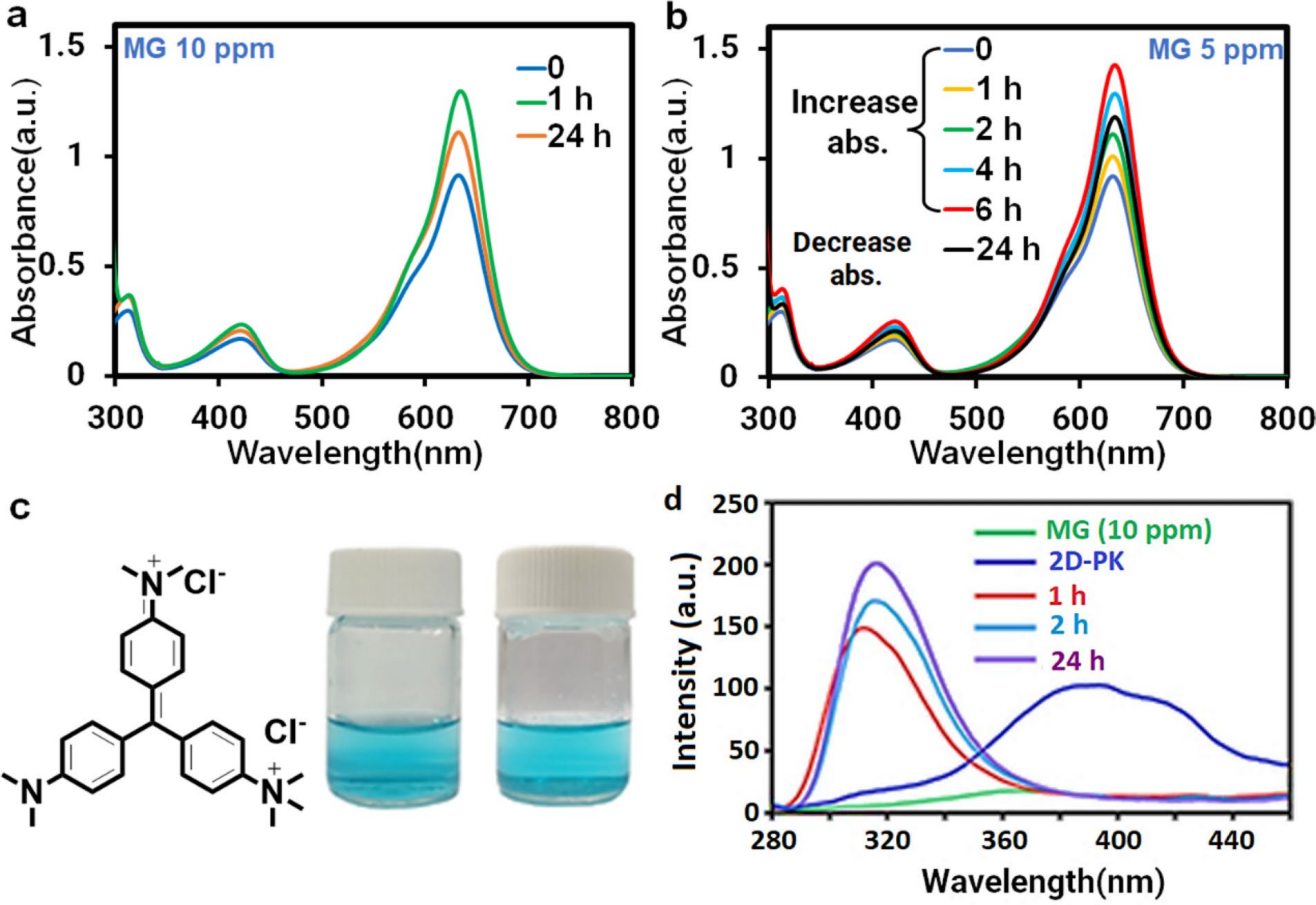
UV–Vis spectra of 10 ppm (**a**) and 5 ppm (**b**) concentrations of aqueous solution of MG before and after incubation with 2D-PK at different time frames. (**c**) Aqueous solution of MG (10 mg/L) before and after incubation with 2D-PK for one month. (**d**) The fluorescence spectra of aqueous solution of MG (10 ppm), before and after incubation with 2D-PK for different time frames, and 2D-PK at λ_ex_ = 270 nm excitation wavelength.

Following structural characterization, the molecular recognition capability of 2D-PK was investigated. A series of anionic and cationic dyes—methyl orange (MO), congo red (CR), rhodamine B (RhB), methyl green (MG), methylene blue (MB), crystal violet (CV), and malachite green (MaG)—were selected as guest molecules and incubated with 2D-PK in aqueous solution. To probe host–guest interactions, UV–Vis absorption spectra of the 2D-PK/dye systems were recorded at different time intervals and dye concentrations (Figs. 4 and 5, S4, and S5).

Among the tested dyes, only CR and MG exhibited pronounced spectral changes upon interaction with 2D-PK (Figs. 4a, b, 5a, b). In the case of CR, the UV–vis absorption intensity decreased significantly following incubation with 2D-PK (Figs. 4a and S6a). This behavior is attributed to nonspecific interactions, such as π–π stacking, which promote aggregation and precipitation of the 2D-PK/CR complexes. At higher dye concentrations, the reduction in absorption intensity became more pronounced, consistent with an increased number of dye molecules interacting with the 2D-PK surface. After 60 min, a red-colored precipitate formed at the bottom of the reaction vessel, indicating extensive aggregation of 2D-PK induced by CR binding (Fig. 4b). SEM and optical microscopy images of the precipitate reveal irregular assemblies composed of multiple stacked sheets (Figs. 4c and S6b).

Additionally, fluorescence spectroscopy shows a red shift of approximately 15 nm for CR upon interaction with 2D-PK, providing further evidence of strong interactions between CR molecules and the 2D-PK sheets (Fig. 4d).

In contrast, the UV absorption of MG increased following incubation with 2D-PK (Fig. 5). At higher MG concentration (10 ppm), UV–Vis absorption initially increased during the first hour of incubation and then decreased with time after 24 h (Fig. 5a). When the dye concentration was reduced to 5 ppm, the UV–Vis absorption continued to rise for a longer period (up to 6 h), but a decrease was observed after 24 h (Fig. 5b). At both concentrations, the initial increase in UV–Vis absorption was attributed to reduced light scattering, resulting from the disassembly of dye aggregates through host–guest interactions^27,28^. In spite of CR, encapsulation of individual MG molecules within specific domains of 2D-PK minimized their aggregation and scattering, leading to enhanced UV–Vis absorption (Figs. 4b and 5a). 2D-PK/MG complex was stable in aqueous solutions, and no precipitation was observed even after a month (Fig. 5c). The fluorescence of 2D-PK/MG complex was investigated, revealing a new emission at 313 nm upon excitation at 244 nm (Fig. 5d). This new emission appeared in a wavelength where no fluorescence for 2D-PK and MG was observed. The emission intensity increased and showed a red shift over a 24-hour period, indicating efficient interaction between MG and 2D-PK. This effect was likely due to the confinement of MG molecules within the 2D-PK sheets, inducing conformational changes and resulting in altered optical behavior^15,29^.

**Fig. 6:**
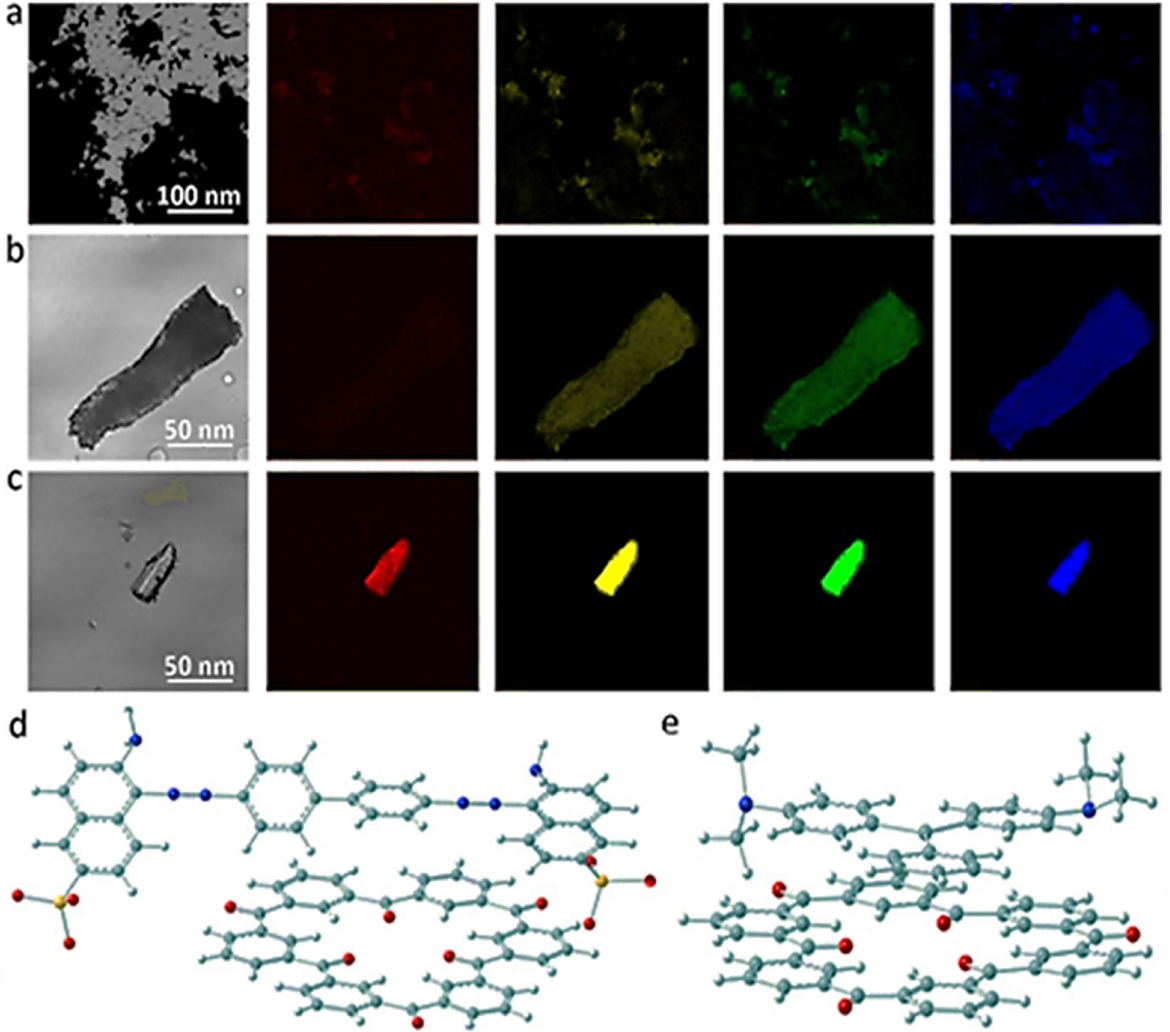
CLSM images of (**a**) 2D-PK sheets, (**b**) free MG, and (**c**) 2D-PK/MG excited at 405 nm (blue), 488 nm (green), 532 nm (yellow) and 635 nm (red). A new fluorescence emission after excitation at 635 nm indicated a specific interaction between dye and 2D-PK and confinement in a special conformation. (**d**) Representation of the optimized conformers for interactions between CR (d) and MG (e) with 2D-PK.

The fluorescence emission of MG, before and after incubation with 2D-PK, was investigated by confocal laser scanning microscopy (CLSM). 2D-PK didn’t show significant fluorescence (Fig. 6a). Free MG showed emission upon excitation at 405 nm, 488 nm, 532 nm and 635 nm (Fig. 6b). Complexation of dye with 2D-PK, however, intensified its emission at different wavelengths dramatically (Fig. 6c). More interestingly a strong red emission was appeared upon excitation at 635 nm. This was a further proof for efficient interactions between MG and 2D-PK in the form charge transfer or confinement individual dye molecules in a special conformation for the more intense or completely new emissions. To gain deeper insights into the origins and nature of molecular recognition, the interaction mechanisms between 2D-PK and various dyes were investigated through computational simulations (Figure S7). A primary objective of these studies was to highlight the pivotal role of π–π interactions in governing the strength and specificity of molecular recognition by 2D-PK.

The calculated adsorption energies (ΔE_ad_) for the interactions between dyes and 2D-PK are summarized in figure S6f. Among different dyes, CR and MG exhibited the highest and lowest adsorption energies, respectively, representing two extremes of interaction strength. The strong π–π interactions between CR and 2D-PK led to a high dye loading and subsequent precipitation of the 2D-PK/CR complex. This phenomenon is the primary reason for the observed decrease in the UV–Vis absorbance of CR. The extended π-conjugated system of CR facilitates strong interactions with multiple sites across one or several 2D-PK sheets simultaneously (Fig. 6d). In contrast, MG showed the weakest ΔE_ad_, suggesting more selective and localized interactions with 2D-PK. Notably, MG was predominantly located within the cavity of 2D-PK, consistent with weaker π–π interactions and more specific binding (Fig. 6e). These defined-site interactions contribute significantly to the molecular recognition behaviour observed in the 2D-PK/MG.

## Discussion

Supramolecular interactions between methyl green and 2D-PK were investigated by ¹H NMR titration. Signals at 8.75 ppm and 7.5–7.6 ppm were assigned to the protons in the ortho and meta position of ^+^NM3 group, respectively.

**Fig. 7:**
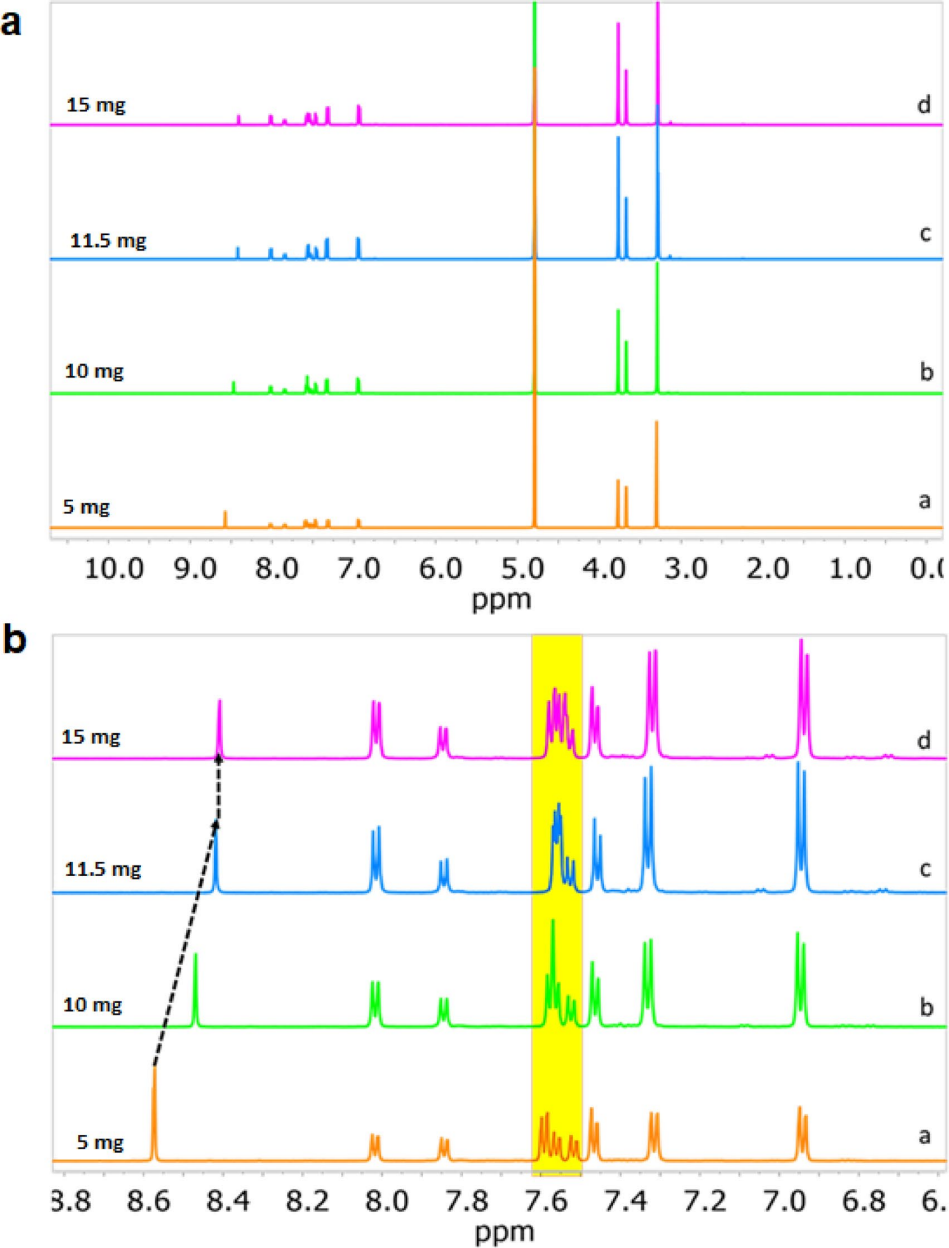
^1^H NMR spectra of MG in the presence of different concentrations of 2D-PK. A clear shift in the signal of ortho proton as well as splitting of meta protons in aromatic ring bearing + NMe₃ versus increasing of the concentration of 2D-PK can be seen.

A progressive downfield shift of the ortho proton together with splitting of the meta proton signal upon increasing 2D-PK concentration suggested efficient supramolecular interactions between methyl green and 2D polymer via ^+^NMe₃ bearing aromatic ring. Such interaction can be specific to methyl green (Fig. 7).

**Fig. 8:**
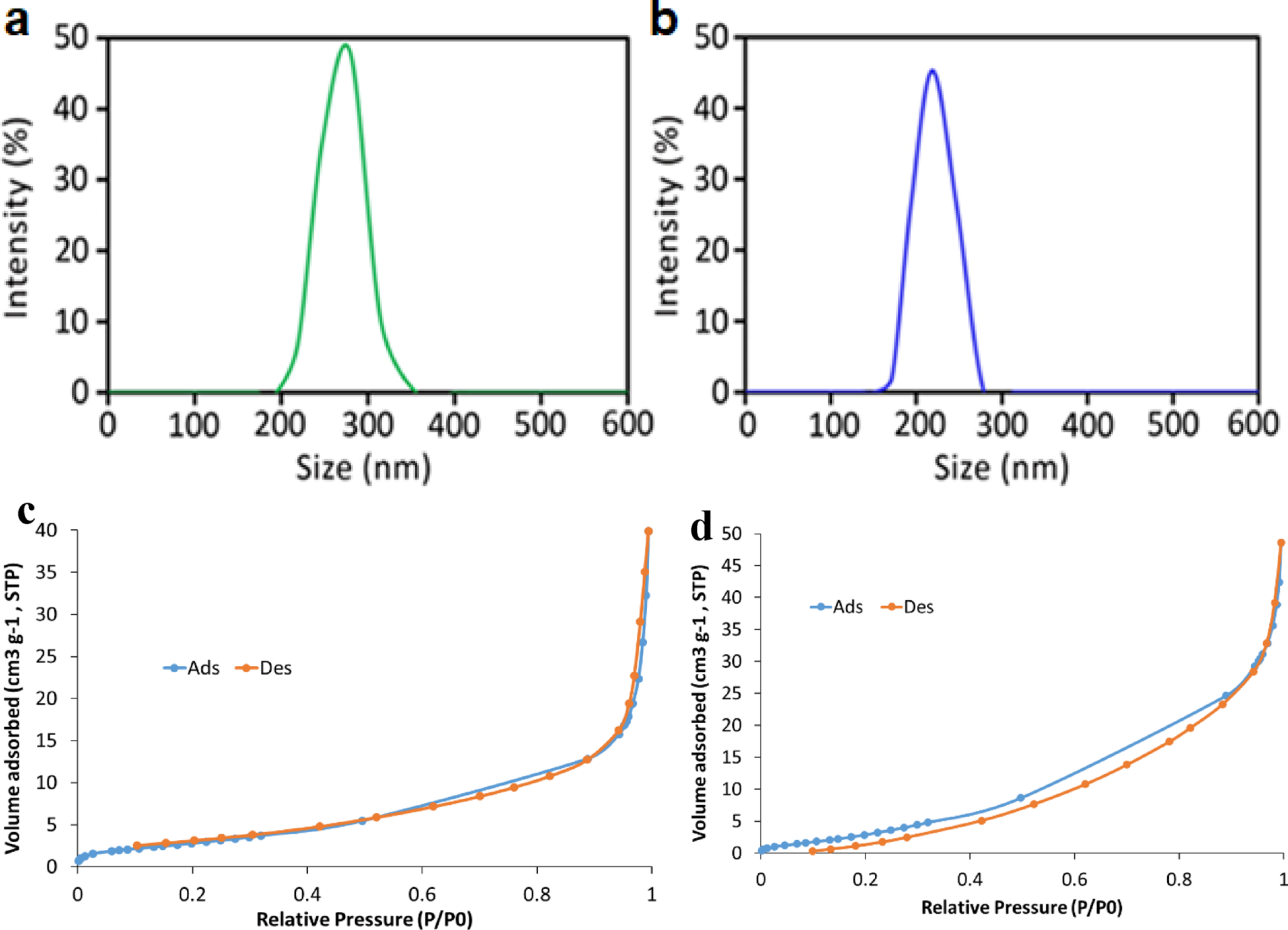
DLS diagrams of MG (10 ppm) before (**a**) and after (**b**) incubation with 2D-PK for 60 min. Nitrogen adsorption/desorption isotherm of the 2D-PK (**c**) and 2D-PK/MG complex.

**Fig. 9:**
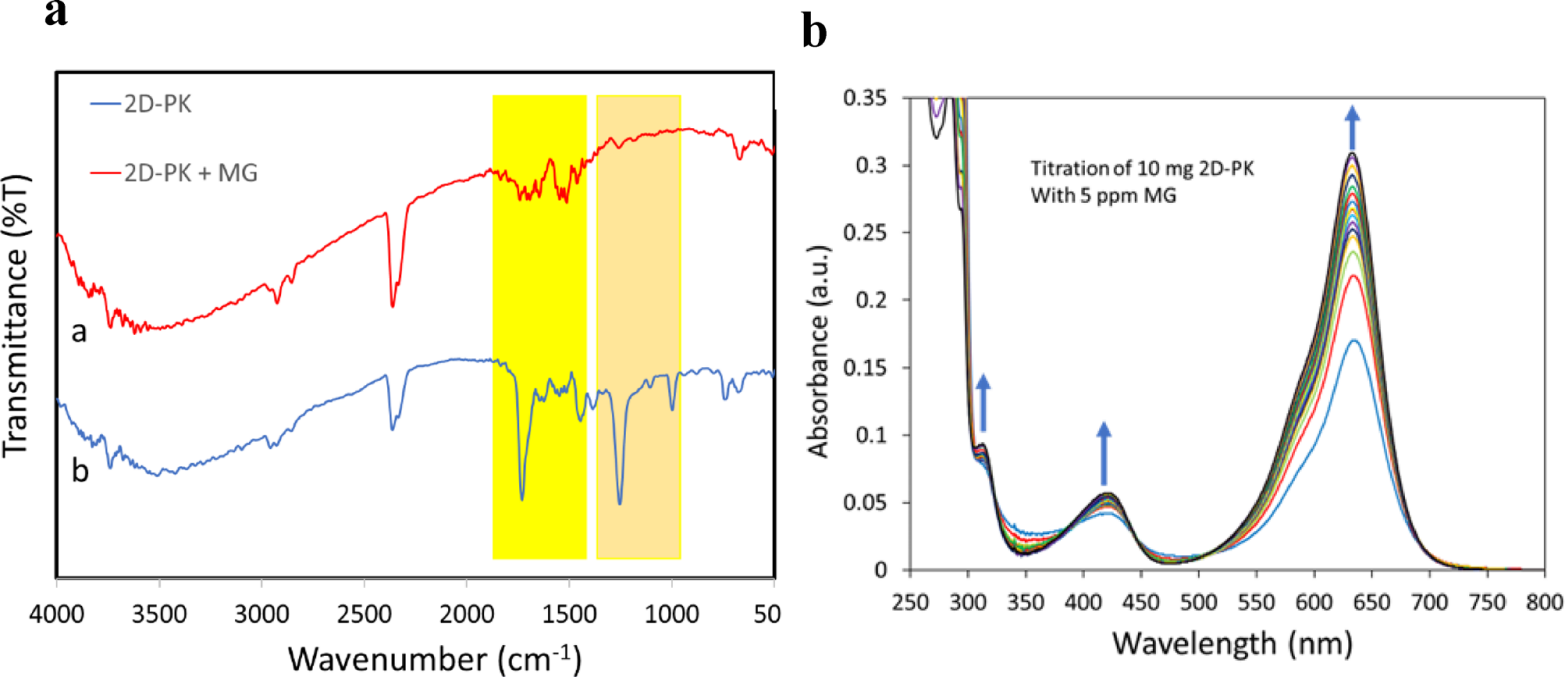
(**a**) FT-IR spectra of 2D-PK before and after addition of 5 ppm MG. (**b**) UV–Visible titration of 1 mg of 2DPK with 10 ppm MG in different volumes over a period of 1–6 h.

Dynamic light scattering (DLS) showed an average size around 280 nm for MG in aqueous solution, indicating large aggregates of this dye (Fig. 8a). After incubation with 2D-PK, however, the size of aggregates reduced to 218 nm (Fig. 8b). This average size belonged to the 2D-PK/MG complex and indicated the ability of 2D-PK sheets for dispersion of MG molecules by specific interactions. To further prove such interactions between methyl green and 2D-PK, BET analysis before and after dye adsorption was conducted. Increasing of the pore volume and surface area of 2D-PK, after dye adsorption, indicated optimal interactions to acquire a higher gas adsorption (Table S1 and Fig. 8c and d).^30^ To further investigate the supramolecular interactions between methyl green and 2D-PK, IR spectra of two-dimensional polymer after and before interaction with dye was recorded. After interaction with methyl green the intensity of carbonyl band decreased dramatically. This could be due to the altered dipole moment upon vibration due to charge transfer (Fig. 9a)^31^. The charge transfer is further proved by appearance a new band at 250–340 nm in UV–Vis spectra (Fig. 9b)^32^.

The loading capacity of 2D-PK was dye-dependent. For CR, precipitation occurred at loadings above 50 ppm per mg of 2D-PK, whereas for MG, loadings as high as 400 ppm were achieved without precipitation (Table S2).

The loading capacity of 2D-PK for MG was evaluated at different pH values (Table S3). In the range of pH 5–8, the loading capacity did not change significantly, suggesting that the supramolecular interactions between the dye and the sheets are not primarily governed by electrostatic effects.

The binding stoichiometry for 2D-PK and MG was determined using UV–Vis spectra and BET analysis (ESI, page S14).

**Fig. 10:**
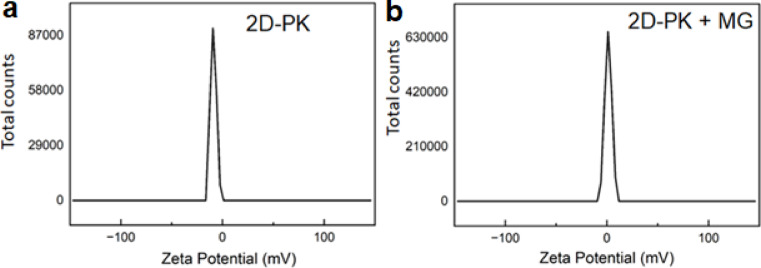
Zeta potential of 2D-PK after and before interaction with MG. Upon binding with MG, the zeta potential shifted from − 15 to + 5 mV.

Zeta potential showed a dramatic change in the surface charge of 2D-PK after interaction with MG (Fig. 10). The negative charge of 2D-PK, due to the carboxyl groups, neutralized after interaction with positively charged MG. Therefore, one of the forces between MG and 2D-PK could be electrostatic interactions.

Nonspecific interactions with most dyes, except MG, lead to aggregation and precipitation. In the case of CR, its larger aromatic backbone may bridge different 2D-PK sheets, resulting in inter-sheet crosslinking and aggregation. In contrast, interactions with MG appear to be more specific and are most likely dominated by ^+^NMe₃ bearing aromatic ring. As a result, the sheets remain dispersed in solution. This behavior suggests that, in the case of MG, the sheets are noncovalently functionalized, leading to enhanced solubility.

The stability of 2D-PK sheets in aqueous solution across different pH values was investigated. 100 mg of 2D-PK was incubated in water at defined pH values at room temperature. After 48 h, any precipitate was collected, and the loss in mass was quantified to determine hydrolyzed material. As shown in Table S4, the change in mass of the initial compound between pH 4 and 9 was minimal, indicating high stability of 2D-PK in this pH range. Partial degradation of the sheets was observed under more acidic or basic conditions.

## Conclusions

In summary, we have developed a crystalline two-dimensional polyketone (2D-PK) through a solid–liquid interfacial Friedel–Crafts acylation carried out on aluminium foil, resulting in micrometer-scale sheets with a highly ordered, layered architecture. Comprehensive characterization using IR spectroscopy, TGA, solid-state NMR, SEM, TEM, AFM, PXRD, and HRTEM confirmed the formation of a structurally well-defined 2D polymer featuring a regular hexagonal network and an interlayer spacing of 3.54 Å. The sharp spectroscopic signatures and narrow thermal decomposition range collectively demonstrate the high degree of structural precision and low defect density attainable through this interfacial synthetic strategy.

Beyond structural elucidation, we uncovered a remarkable degree of selective molecular recognition imparted by 2D-PK. Among a diverse series of cationic and anionic dyes, methyl green (MG) displayed uniquely strong and specific interactions with the polymer, even at low concentrations (5–10 ppm). These interactions gave rise to substantial modulation of fluorescence behaviour, including pronounced enhancement and red-shifted emission features that do not appear in the free dye or in 2D-PK alone. Supporting studies, such as NMR titration, DLS, BET analysis, IR spectroscopy, zeta potential measurements, and computational modelling, indicate that the binding selectivity arises from a synergistic combination of electrostatic attraction, confinement of MG within the polymer’s cavities, and charge-transfer-assisted stabilization. These supramolecular effects not only alter the optical response of MG but also suggest a high degree of shape, size, and charge complementarity between the dye and the 2D-PK framework.

Taken together, our findings highlight 2D-PK as a promising platform for chemical sensing applications based on fluorescence modulation and selective host–guest interactions. The ability of this material to selectively recognize molecular species in aqueous media, while simultaneously amplifying or shifting their emission profiles, opens opportunities for its integration into responsive optical devices, environmental monitoring systems, and bioanalytical technologies. More broadly, this work demonstrates the potential of interfacial Friedel–Crafts chemistry as a versatile route to structurally controlled 2D polymers with tunable physicochemical properties. Continued exploration of this synthetic strategy may enable the design of next-generation 2D materials with engineered pore architectures, enhanced stability, and tailored affinity toward specific molecular targets, thereby expanding the scope of 2D polymers in supramolecular chemistry, sensing, and optoelectronic applications.

## Methods

The Fourier transform infrared (FTIR) spectra were recorded at ambient temperature using a Shimadzu FT-IR 8400 spectrometer. The measurements were conducted with a KBr pellet, prepared at a weight ratio of 5 to 200 mg, covering a scanning range from 4000 to 400 cm^−1^. The Ultraviolet-Visible (UV–Vis) spectra were recorded using a Shimadzu UV–Vis 1650 PC spectrophotometer, which was equipped with a quartz cell featuring a 1.0 cm path length. The absorption spectra of the samples were measured at ambient temperature^13^. C Solid-state cross-polarization magic-angle spinning (CP/MAS) NMR spectra were acquired using a JEOL ECZ600 MHz spectrometer, functioning at a frequency of 150.9 MHz. Scanning electron microscopy (SEM) images were acquired using a TESCAN SEM instrument from Berno, Czech Republic, operated under vacuum conditions at a voltage of 10 kV. A water dispersion of the samples (0.1 mg/mL) was deposited onto a grid, followed by solvent evaporation at ambient temperature. The dried samples were then coated with a thin gold layer via sputtering for 15 s before SEM imaging. The elemental composition and mapping of 2D-PK were assessed using energy-dispersive X-ray spectroscopy (EDX), conducted via scanning electron microscopy (SEM) using a TESCAN instrument (Brno, Czech Republic). The analysis was performed under vacuum conditions at an operating voltage of 10 kV, with an integrated energy-dispersive X-ray spectrometer. Powder X-ray diffraction (P-XRD) patterns of 2D-PK were obtained using a STADIP diffractometer from the German company STOE. The measurements were performed with Cu-Kα radiation at an operating voltage of 35 kV, covering a scan range of 2θ from 5° to 100°. High-resolution transmission electron microscopy (HRTEM) images were acquired using a TEC9G20 instrument from FEI Company (USA), operated under vacuum with a maximum accelerating voltage of 200 kV. TEM samples were prepared by applying a water dispersion of 2D-PK (0.2 mg/mL) onto a grid, followed by vacuum drying. Confocal laser scanning microscopy (CLSM) images were captured using a Leica apparatus (TCS SPE) with the layered photography method (Z-Scan), featuring a maximum scan size of 2 μm at ambient temperature. To prepare a confocal sample, a solution containing 2D-PK (0.3 mg/mL) and the desired dye was prepared. After 24 h, solution was dropped on grid and used for imaging. Optical microscopy samples were prepared by dropping 2D-PK water solution (0.1 mg. mL^− 1^) onto a silica grid that was about 1 mm thick, then subjecting it to vacuum-drying. Dynamic Light Scattering (DLS) measurements were carried out using the Zeta Potential Analyzer and SZ-100z Dynamic Light Scattering device, manufactured by Horiba Jobin Yvon. The nitrogen adsorption desorption isotherms and properties such as specific surface area and pore volume of the magnetic nanocomposite were measured using the BET (Micromeritics TriStar II plus) method. Thermogravimetric analysis (TGA) was recorded by STA PT1600 Linseis (Robbinsville, USA), (Netzsch TG 209 f1 lris) under nitrogen at range 25 °C to 800 °C range with 10 °C /min heating rate. Dye (10 ppm) and 2D-PK (1 mg) were mixed in aqueous solution and stirred for 24 h. Then, solvent was evaporated and the remained materials were used for TGA measurement. X-ray photoelectron spectroscopy (XPS) was recorded by dispersing sample in methanol and evenly distributed dropwise across the surface of gold substrates. XPS spectra were recorded using a Kratos Axis Ultra DLD spectrometer equipped with a monochromatized Al Kα X-ray source (1486.69 eV) using an analyzer pass energy of 80 eV for survey spectra that were used for quantification. High-resolution, core-level O1s and C1s were recorded in FAT (fixed analyzer transmission) mode at a pass energy of 20 eV. Both the electron emission angle and the source-to-analyzer angle were 60°. The binding energy scale of the instrument was calibrated following a Kratos Analytical Ltd procedure that used ISO 15,472 binding energy data. Spectra were recorded by setting the instrument to the hybrid lens mode and the slot mode, which provided approximately a 300 × 700 µm^2^ analysis area and using charge neutralization. All XPS spectra were processed with the UNIFIT program (version 2022). A Gaussian/Lorentzian product function peak shape model GL (30) was used in combination with a Shirley background. If not otherwise denoted, the L-G mixing for component peaks in all spectra were constrained to the value of 0.37. Peak fitting of C1s spectra was performed by using a symmetric peak shape model for all component peaks. After peak fitting of the C1s spectra, all the binding energies were calibrated in reference to the aliphatic C1s component at a binding energy of 285.0 eV. Atomic force microscopy in tapping mode was performed in 512 pixels resolution at room temperatures. Samples were prepared by drop casting an aqueous dispersion of 2D-PK on silica and evaporation of water at room temperature.

### NMR titration was performed as following

NMR measurements were performed as follows: A suspension containing 10 mg of polymer and 10 mL of deuterium oxide (D₂O) was prepared. For each sample, 1 mL of the stock solution was withdrawn using a syringe and a specified amount of methyl green dye (5 mg, 10 mg, 11.5 mg, 15 mg) was dissolved in it before being transferred to the NMR tube. The NMR spectra of each were then recorded separately. The NMR spectra of samples were recorded by a Jeol ECX 400 spectrometer or on a Bruker BioSpin Avance 700 spectrometer (Bruker Corporation, Billerica, MA, USA) (at 295 K). Chemical shifts were reported in ppm using the deuterated solvent peak as the internal standard and tetramethyl silane (SiMe4) was used for internal calibration at 125 MHz with complete proton decoupling.

### Synthesis of two-dimensional polyketone (2D-PK)

First, an aluminum foil with 1 cm ×1 cm × 0.005 mm dimensions was washed by distilled water and ethanol and fixed in a 25 mL single-neck reaction flask and heated in an oil bath at 150 °C under Argon for 24 h. Then, temperature was reduced to 40 °C and a solution of 1,3,5-benzenetricarbonyltrichloride (1.88mmol, 0.5 g) in 1,2-dichloroethane (5 mL) was added to the reaction flask. Afterwards, mesitylene (2.49 mmol, 0.3 g) was added to the reaction flask and stirred at 40 °C under argon for 24 h slowly. In the next step, the aluminum foils were taken out from reaction flask and washed by methanol, acetone, ethanol and water solvents and the foils transferred to another container. Then, solvent was evaporated under vacuum at room temperature and product was dialyzed against water and methanol for 48 h. The purified product as a white solid was collected after lyophilization.

### Conditions for obtaining UV–visible spectra of the polymer before and after dye absorption

The UV–Visible spectrum of the two-dimensional polymer was performed by first pouring 1 mg of the 2D-PK into distilled water and then sonicating it for 15 min using an ultrasonic device. This allowed the 2D polymer to be removed from the aggregated state and become uniformly dispersed in water. Then, the UV–Visible spectrum was immediately taken.

## Supplementary Information

Below is the link to the electronic supplementary material.

Supplementary Material 1

## Data Availability

The datasets used and/or analysed during the current study available from the corresponding author on reasonable request.
