# Supplementary material for: Supramolecular recognition on the surface of two-dimensional polyketones
Source: Sci Rep. 2026 Apr 17;16:18206. doi: 10.1038/s41598-026-41144-8 (PMC13261043; doi:10.1038/s41598-026-41144-8)
Supplement: Supplementary file 1 — Supplementary Material 1 [file 41598_2026_41144_MOESM1_ESM.docx]

Supporting Information

Supramolecular Recognition on the Surface of Two-Dimensional Polyketones

*Sanaz kazemi^a^, Mohsen Adeli^a^*, Mohammad Nemati^a^*

*^a^Faculty of Science, Department of Chemistry, Lorestan University, Khorramabad, Iran,*

**Corresponding author: adeli.*[*m@lu.ac.ir*](mailto:m@lu.ac.ir)

Contents

[Materials and methods 2](#_Toc212495718)

[Materials 2](#_Toc212495719)

[Characterization 2](#_Toc212495724)

[**Figure S1.** XPS spectra of 2D-PK. 3](#_Toc212495726)

[**Figure S2.** SEM image of the surface of aluminum before and after Fridel-Crafts reaction. ……5](#_Toc212495727)

[**Figure S3.** AFM images of 2D-PK indicated flat stacked sheets. 5](#_Toc212495730)

[**Figure S4.** UV-vis spectra of aqueous solution of dyes before and after incubation with 2D-PK at different time frames. 6](#_Toc212495731)

[**Figure S5.** UV-vis of dyes before and after incubation with 2D-PK for 24 h. 7](#_Toc212495732)

[**Figure S6.** UV-vis spectra of aqueous solutions of CR with 10 ppm concentration before and after incubation with 2D-PK at different time frames. Also, optical microscopy image of 2D-PK sheets after incubation with CR for 24 h. 8](#_Toc212495733)

[**Table S1.** Porous properties of 2D-PK adsorbent using BET analysis before and after MG dye adsorption. 13](#_Toc212495736)

[**Figure S7.** Representation of the optimized conformers for interactions between dyes and 2D-PK. 9](#_Toc212495743)

## **Table S1.** Porous properties of 2D-PK adsorbent using BET analysis before and after MG dye adsorption……………………………………………………………………………………….10

**Table S2.** Maximum loading capacity (ppm dye/mg 2D-PK), before precipitation for different dyes………………………………………………………………………………………………12

**Table S3.** Loading capacity (ppm MG/mg 2D-PK), before precipitation in different pH……….13

**Table S4.** Weight loss (%) of 2D-PK after incubation in water with different pH for two dyes..13

[**References** 13](#_Toc212495744)

# Materials and methods

# Materials

1,3,5-Benzenetricarbonyl trichloride, mesitylene, dialysis tube (benzylated tube MWCO: 2000), methyl orange (MO), rhodamine B (Rh B), methyl green (MG), congo red (CR), methylene blue (MB), crystal violet (CV), and malachite green (MaG) were purchased from Sigma-Aldrich (Schnelldorf, Germany). 1,2-Dichloroethane, methanol, acetone and ethanol, were purchased from Merck (Germany). Aluminum foil was purchased from Third Millennium Aluminum Factory (Iran).

# Characterization

# The structure of 2D-PK was further investigated by XPS.

Carbon and oxygen were elements that were detected in the Survey spectra. In C1s spectra, clear peak components for C=C and C=O bonds can be seen. In O1s spectra peak components related to carbonyl and carboxyl groups are observed.

**
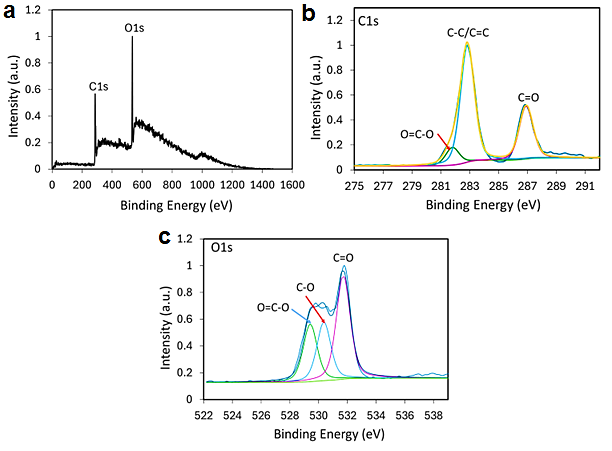
**

## **Figure S1.** XPS spectra of 2D-PK. a) Survey spectra indicated carbon and oxygen as the main components for 2D-PK. b) C1s spectra showed peak components for carbonyl and C=C bonds. c) O1s spectra clearly showed two peak components for carbonyl and carboxyl groups.


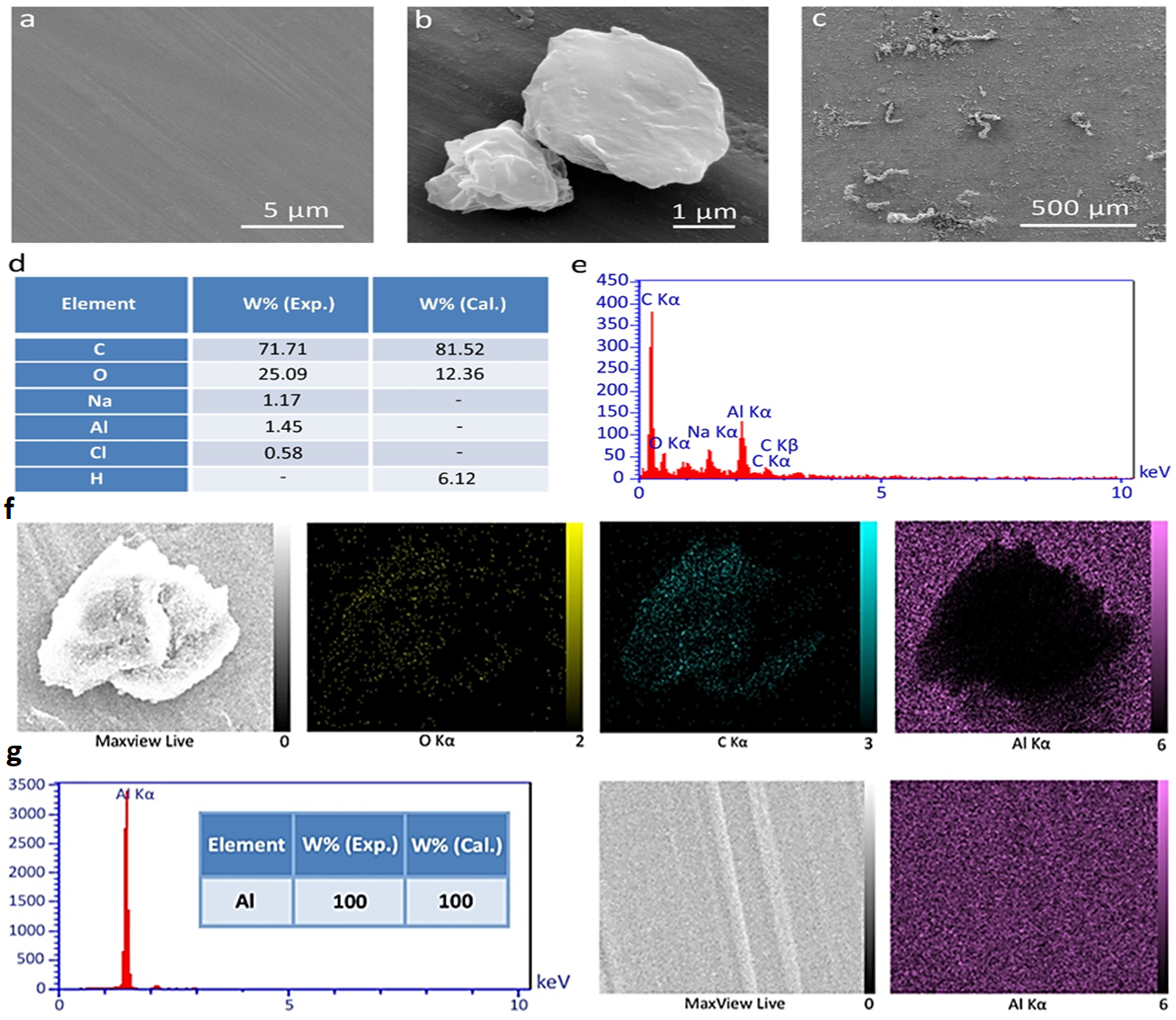


## **Figure S2.** SEM image of the surface of aluminum a) before and b) after Fridel-Crafts reaction. c) Worm-like objects consisting stacked layers of 2D-PK with several hundred micrometers length emerged on aluminum. d) The composition and e) elemental distribution of 2D-PK using Energy Dispersive X-ray Spectroscopy (EDX). e) EDX analysis of 2D-PK, indicating carbon and oxygen as the main elements, in agreement with theoretical values. f) Elemental mapping of a 2D-PK sheet on the aluminium surface. EDX analysis of the surface of aluminium before Friedel-Crafts reaction shows only Al element (bottom row).

# TGA thermogram of 2D-PK after dyes adsorption

TGA thermogram of 2D-PK after dyes adsorption was investigated. While methylene blue and Congo Red didn’t change the main weight loss of 2D-PK (250-350 ℃), it was changed into a broad weight loss from 200 ℃ to 650 ℃. This indicated a different interaction between MG and 2D-PK in comparison with other dyes.


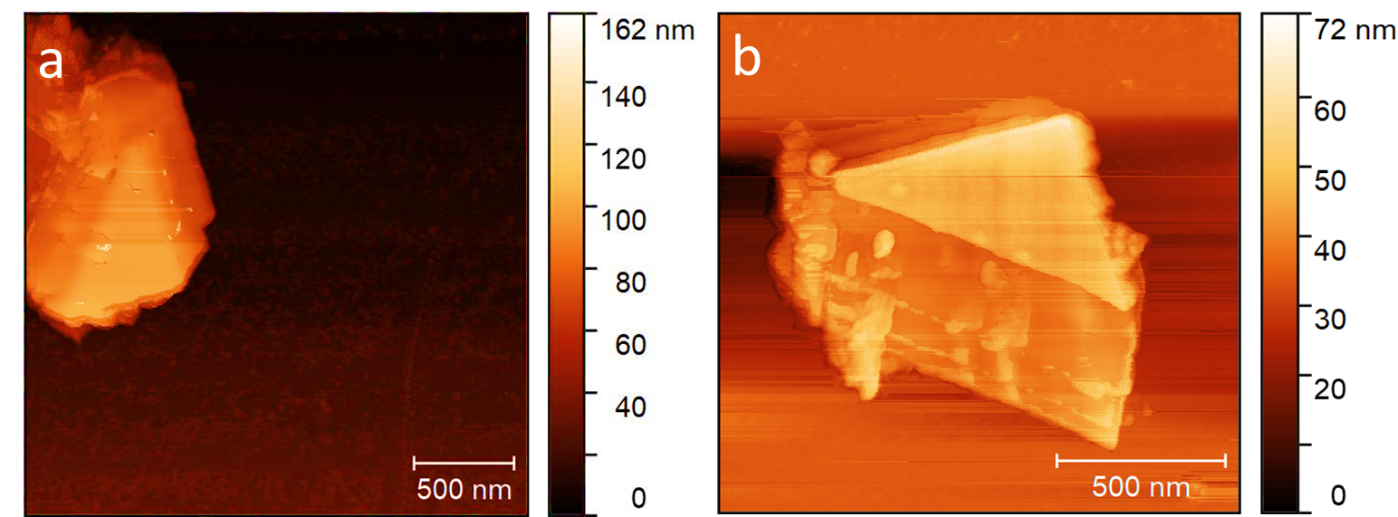


## **Figure S3.** AFM images of 2D-PK indicated flat stacked sheets.


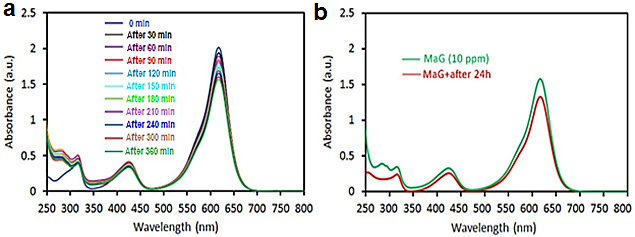


## **Figure S4.** a) UV-vis spectra of aqueous solution of malachite green (10 ppm) before and after incubation with 2D-PK at different time frames. [b) UV-vis of MG before incubation and after incubation with 2D-PK for 24 h](https://bing.com/search?q=translate+Persian+to+English+%d8%b7%db%8c%d9%81+%db%8c%d9%88%d9%88%db%8c+%d8%b1%d9%86%da%af+%da%a9%d9%86%da%af%d9%88%d8%b1%d8%af+%d8%a7%d8%b2+%d8%b2%d9%85%d8%a7%d9%86+%d8%b5%d9%81%d8%b1+%d9%88+%d8%a8%d8%b9%d8%af+%d8%a7%d8%b2+24+%d8%b3%d8%a7%d8%b9%d8%aa). A slight decrease in the UV-vis adsorption of MaG after 24 hours was assigned to the adsorption of a small amount of dye on 2D-PK and precipitation of 2D-PK/MaG.


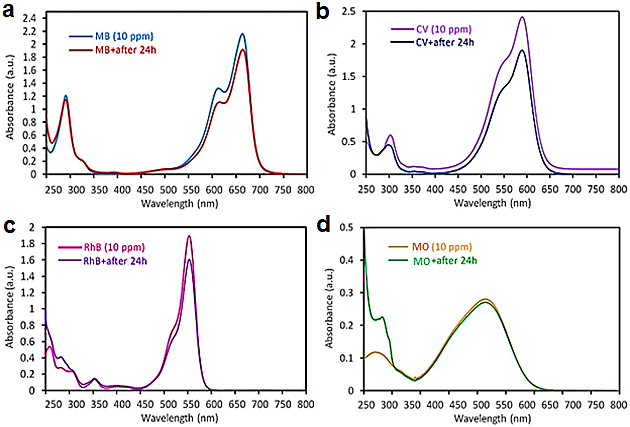


## **Figure S5.** [UV-vis of MB (a), CV (b), Rh B (c), MO (d) before and after incubation with 2D-PK for 24 h](https://bing.com/search?q=translate+Persian+to+English+%d8%b7%db%8c%d9%81+%db%8c%d9%88%d9%88%db%8c+%d8%b1%d9%86%da%af+%da%a9%d9%86%da%af%d9%88%d8%b1%d8%af+%d8%a7%d8%b2+%d8%b2%d9%85%d8%a7%d9%86+%d8%b5%d9%81%d8%b1+%d9%88+%d8%a8%d8%b9%d8%af+%d8%a7%d8%b2+24+%d8%b3%d8%a7%d8%b9%d8%aa). A slight decrease in the UV-vis adsorption of dyes after 24 hours was assigned to the adsorption of a small amount of dye on 2D-PK and precipitation of 2D-PK/MB, CV, RhB, and MO.


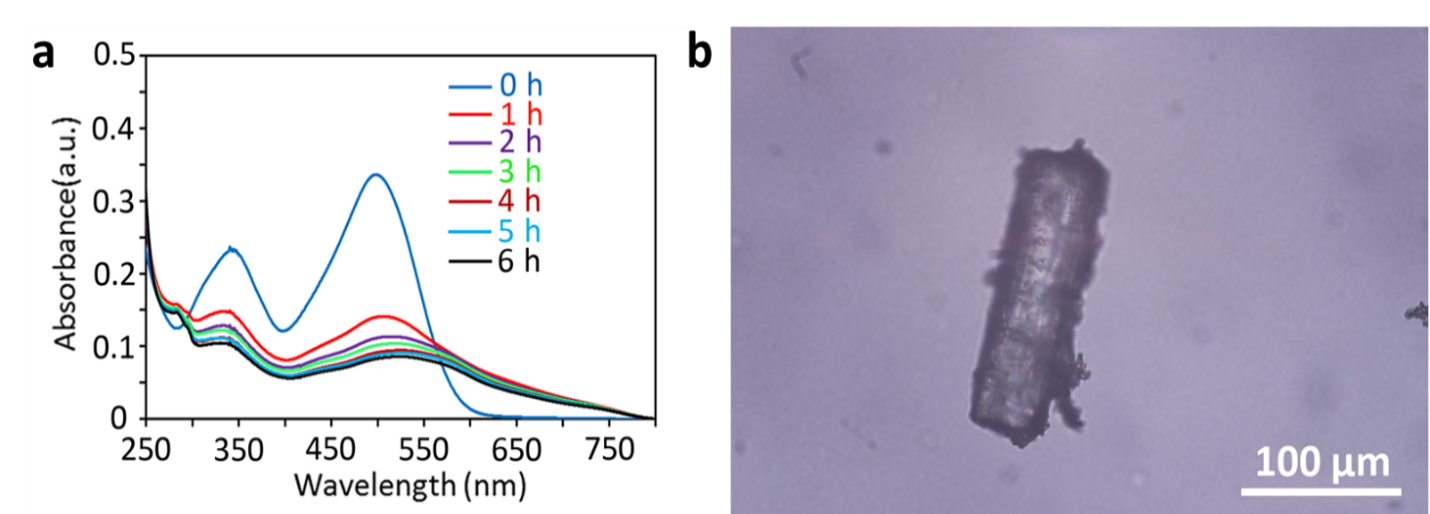


## **Figure S6.** a) UV-vis spectra of aqueous solutions of CR with 10 ppm concentration before and after incubation with 2D-PK at different time frames. b) Optical microscopy image of 2D-PK sheets after incubation with CR for 24 h.

**
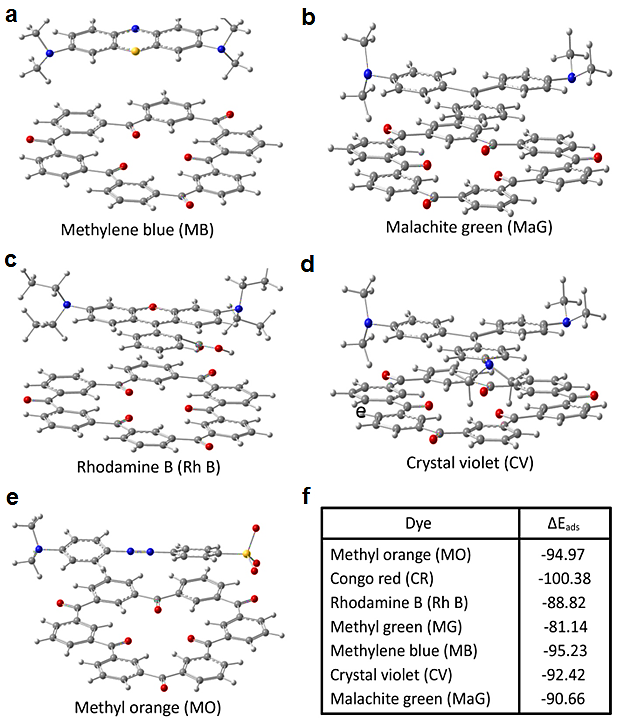
**

## **Figure S7.** a-e) Representation of the optimized conformers for interactions between methylene blue, malachite green, rhodamine B, crystal violet, and methyl orange with 2D-PK. ^2^ f) Adsorption energies (ΔEads) for interactions between different dyes and 2D-PK, computed at the M05-2X/6-31G** level of theory.

## **Table S1.** Porous properties of 2D-PK adsorbent using BET analysis before and after MG dye adsorption.

|  | **BET Surface Area** | **BJH Adsorption cumulative volume of pores** | **average pore diameter**  **(4V/A by BET)** |
| --- | --- | --- | --- |
| **Polymer** | 11.4519 m²/g | 0 .070196 cm³/g | 8.92907 nm |
| **Polymer +MG (10ppm)** | 18.4319 m²/g | 0 .088101 cm³/g | 10.03054 nm |

# The mechanism of interactions between methyl green and 2D-PK

As shown by NMR titration (mentioned above), one of the main structural factors leading to specific interactions between methyl green and 2D-PK is phenyl ring bearing ^+^NMe₃ functional group.

To further prove such interactions between methyl green and 2D-PK, BET analysis before and after dye adsorption were conducted. According to the data obtained from the BET analysis the pore volume and surface area of the polymer has increased after dye adsorption. This is due to a structural change induced by the adsorption process known as breathing. This is owing to the optimization of these types of materials to acquire higher adsorption capacity after the first interaction with the target molecule.

According to the diagrams and data obtained from the BET analysis, it can be seen that isotherm is of the VI types and its hysteresis is of the H3 type. This type of hysteresis loop is indicative of the mesoporous character of the adsorbent.

This paradoxical phenomenon is actually one of the key and interesting properties of some 2D polymers and metal-organic frameworks (MOFs) or covalent-organic polymers (COFs). This can be explained by several mechanisms:

Main mechanism: “Breathing” or host-guest induced phase transition

This is the main reason for such a phenomenon. In this mechanism, the dye adsorption e trigger a large-range structural optimization in the polymer network as below:

i) Initial compact state:

The 2D polymer structure in the “dry” state or without adsorbing a substance (water, solvent or in this case, dye) may be is in a “closed-pore” or “semi-open” phase. In this state, the polymer sheets or layers are packed and the potential pores are not fully open. As a result, the volume of pores and the available surface area are low.

ii) Induction of phase transition by dye molecules:

When dye molecules (guests) start to penetrate and adsorb into small pores or on primary surfaces, host-guest interactions are established. These interactions can include hydrogen bonding, π-π interactions, van der Waals interactions, or even electrostatic. These interactions provide the energy needed to overcome the energy barrier between the compact and open phases. Dye molecules act like a "wedge" and, by applying force, push the polymer sheets apart and unlock the structural lock.

iii) Transition to an open and swollen phase:

A cooperative phase change occurs in the entire structure. The polymer network changes from a compacted state to an “open-pore” or “swollen” state. In the new phase, the volume of the pores increases because the spacing between the layers or structural units increases.^1^

**Binding stoichiometry**

UV-visible titration of 1 mg of 2DPK with 5 ppm MG in different volumes was carried out for a period of 1-6 hours. 2D-PK (1 mg) was poured into a beaker and MG (3 ml) of 5 ppm stock solution was added to it each time and its spectrum was recorded after 20 minutes of shaking. Absorption peak at 285 nm related to MG decreased addition of MG. However, the intensity of peaks at 315 nm, 430 nm and 640 nm increased by addition of each volume of MG. These changes continued until addition of 40-50 ml of dye solution, where any significant change in the intensity of peaks was not detected.

These spectral changes in the absorption spectrum of MG indicated supramolecular interactions including π-π stacking, electrostatic interactions and charge transfer between MG and the 2D-PK.

According to the 2D-PK titration diagram with MG at a concentration of 5 ppm, it can be found that the volume of polymer saturation by methyl green dye is 50 ml.

As a result, the mass of methyl green dye is equal to:

C*V=molMG

mol MG= 0.005g/ml* 50ml= 0.25 mg=2.5*10^-4^g

As a result, the number of moles of methyl green is equal to:

2.5*10^-4^g/458.47g.mol^-1^= 5.45* 10^-7^ mol

Number of dye molecules (number of MG molecules added):

N=5.45* 10^-7^ mol* 6.022*10^23^= 3.28*10^17^ molecules

On the other hand, the total surface area of ​​the polymer based on BET analysis is equal to:

18 m^2^.g^-1^ * 0.001g= 0.018 m^2^ => A total

And the area of ​​one methyl green molecule is equal to 1 nm^2^, which is equal to 10^-18^ m^2^.

As a result, the number of molecules that can cover the polymer surface is equal to:

Nmax= ATotal/AMG= 10^-18^ m^2^/0.018 m^2^=1.8*10^16^ molecules

The ratio of added molecules to adsorbed molecules:

Ntotal,MG/Nmax = 3.28*10^17^/1.8*10^16^ = 19.2

The average area of ​​each sheet based on SEM images is equal to:

(4μm*3μm)= 12μm^2^ = 1.2*10^-11^ m^2^

The number of molecules that can be adsorbed by MG dye for each sheet is equal to:

Nsheet=Asheet/AMG= 1.2*10^-11^ m^2^ /10^-18^ m^2^=1.2*10^7^ Molecules.

The calculated adsorption energies (ΔE_ad_) for the interactions between dyes and 2D-PK are summarized in figure S15f. Among different dyes, CR and MG exhibited the highest and lowest adsorption energies, respectively, representing two extremes of interaction strength. The strong π–π interactions between CR and 2D-PK led to a high dye loading and subsequent precipitation of the 2D-PK/CR complex. This phenomenon is the primary reason for the observed decrease in the UV-vis absorbance of CR. The extended π-conjugated system of CR facilitates strong interactions with multiple sites across one or several 2D-PK sheets simultaneously (Figure 5d). In contrast, MG showed the weakest ΔE_ad_, suggesting more selective and localized interactions with 2D-PK. Notably, MG was predominantly located within the cavity of 2D-PK, consistent with weaker π–π interactions and more specific binding.

**Table S2.** Maximum loading capacity (ppm dye/mg 2D-PK), before precipitation for different dyes.

| Dye | MO | CR | RhB | MG | MB | CV | MaG |
| --- | --- | --- | --- | --- | --- | --- | --- |
| Loading capacity | 10 ppm | 50 | 15 | 400 | 30 | - | 27 |

**Table S3.** Loading capacity (ppm MG/mg 2D-PK), before precipitation in different pH……..12

| pH | 2 | 3 | 4 | 5 | 6 | 7 | 8 | 9 | 10 |
| --- | --- | --- | --- | --- | --- | --- | --- | --- | --- |
| Loading capacity | - | - | 20 | 300 | 300 | 400 | 330 | 45 | - |

**Table S4.** Weight loss (%) of 2D-PK after incubation in water with different pH for two dyes.

| pH | 2 | 3 | 4 | 5 | 6 | 7 | 8 | 9 | 10 |
| --- | --- | --- | --- | --- | --- | --- | --- | --- | --- |
| Weight loss | 23% | 22% | 5% | 5% | 4% | 4% | 3% | 5% | 10% |

# References

(1) Alhamami, M.; Doan, H.; Cheng, C.-H. A review on breathing behaviors of metal-organic-frameworks (MOFs) for gas adsorption. *Materials* **2014**, *7* (4), 3198-3250. Fernández-Seriñán, P.; Roztocki, K.; Safarifard, V.; Guillerm, V.; Rodríguez-Hermida, S.; Juanhuix, J.; Imaz, I.; Morsali, A.; Maspoch, D. Modulation of the Dynamics of a Two-Dimensional Interweaving Metal–Organic Framework through Induced Hydrogen Bonding. *Inorganic chemistry* **2024**, *63* (12), 5552-5558. Ghoufi, A.; Benhamed, K.; Boukli-Hacene, L.; Maurin, G. Electrically induced breathing of the MIL-53 (Cr) metal–organic framework. *ACS central science* **2017**, *3* (5), 394-398. Durán-Egido, V.; García-Giménez, D.; Martínez-López, J. C.; Pérez-Vidal, L.; Carretero-González, J. Metal–organic frameworks as fillers in porous organic polymer-based hybrid materials: innovations in composition, processing, and applications. *Polymers* **2025**, *17* (14), 1941. Graham, A. J.; Allan, D. R.; Muszkiewicz, A.; Morrison, C. A.; Moggach, S. A. The Effect of High Pressure on MOF‐5: Guest‐Induced Modification of Pore Size and Content at High Pressure. *Angewandte Chemie International Edition* **2011**, *50* (47), 11138-11141. Thapa, K. B.; Lee, S.-Y.; Park, S.-J. Diversified functional applications of flexible metal-organic frameworks. *Materials Today Advances* **2025**, *26*, 100588.

(2) Becke, A. D. Density‐functional thermochemistry. III. The role of exact exchange. *The Journal of chemical physics* **1993**, *98* (7), 5648-5652. Zhao, Y.; Truhlar, D. G. Assessment of model chemistries for noncovalent interactions. *Journal of Chemical Theory and Computation* **2006**, *2* (4), 1009-1018. Boys, S. F.; Bernardi, F. The calculation of small molecular interactions by the differences of separate total energies. Some procedures with reduced errors. *Molecular physics* **1970**, *19* (4), 553-566. Schmidt, M. W.; Baldridge, K. K.; Boatz, J. A.; Elbert, S. T.; Gordon, M. S.; Jensen, J. H.; Koseki, S.; Matsunaga, N.; Nguyen, K. A.; Su, S. General atomic and molecular electronic structure system. *Journal of computational chemistry* **1993**, *14* (11), 1347-1363.
